# Supplementary figures and images for: Heterologous expression and characterization of mutant cellulase from indigenous strain of Aspergillus niger
Source: PLoS One. 2024 May 15;19(5):e0298716. doi: 10.1371/journal.pone.0298716 (PMC11095671; doi:10.1371/journal.pone.0298716)

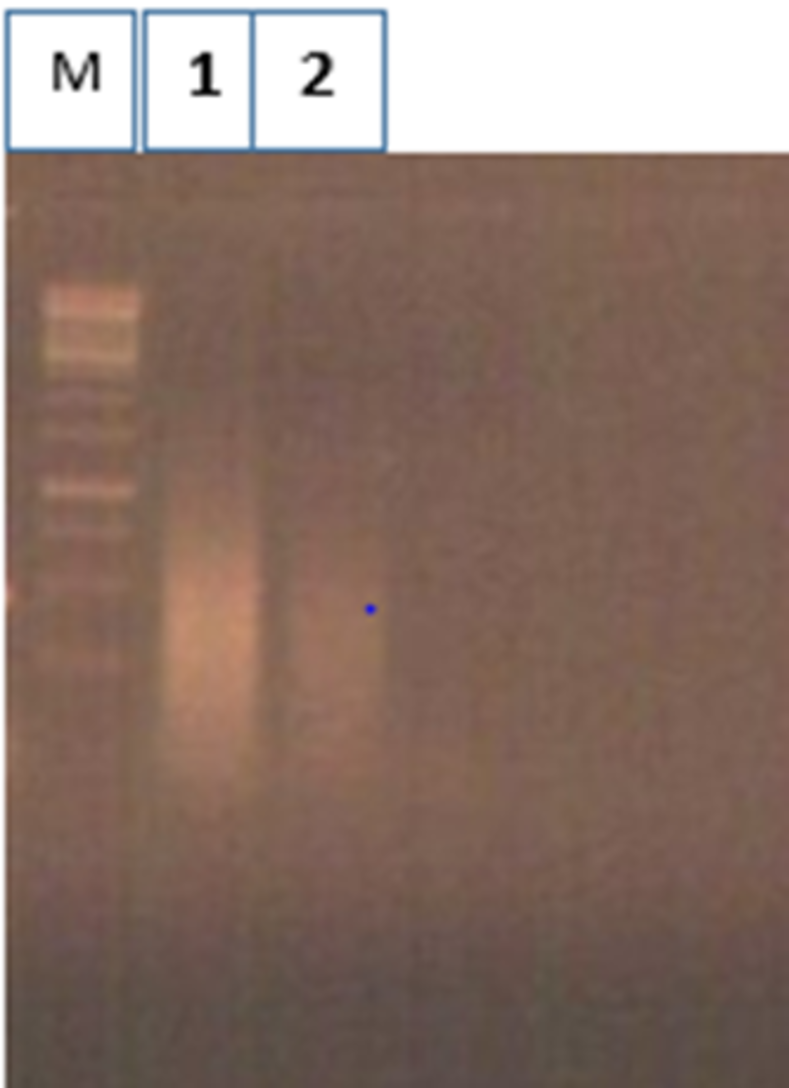

Supplement: S1 Fig — Lane M represents 1 Kb DNA ladder (Invitrogen). Lane 1 represents mRNA of native and Lane 2 represents mRNA of mutant strain of A. Niger. (TIF) [file pone.0298716.s001.tif]

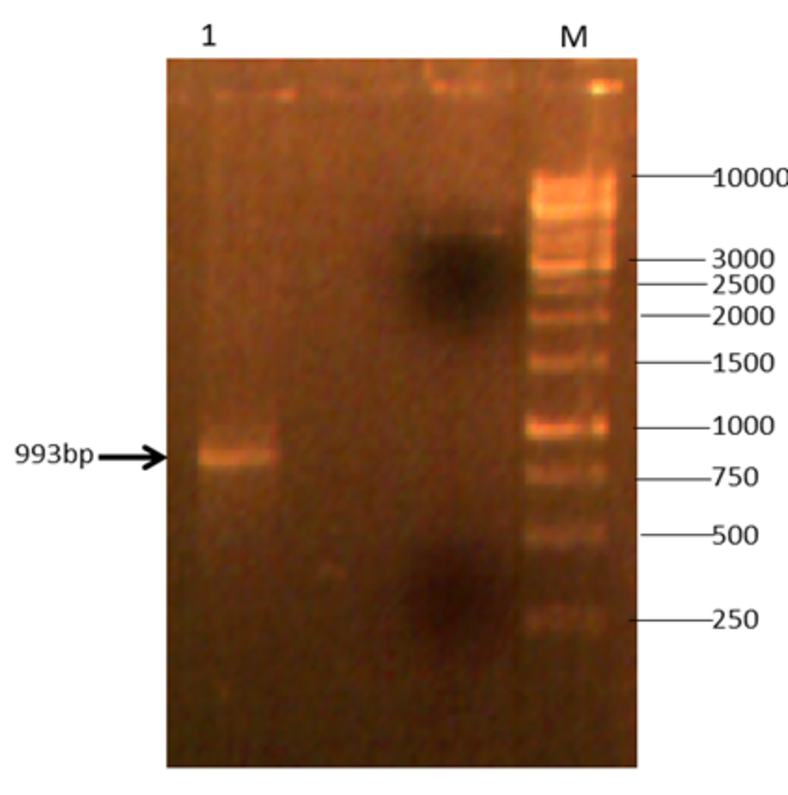

Supplement: S2 Fig — Lane M represents Gene Ruler 1 kb DNA ladder for size comparison of amplified gene and Lane 1 represents DNA band of native endoglucanase B (993bp). (TIF) [file pone.0298716.s002.tif]

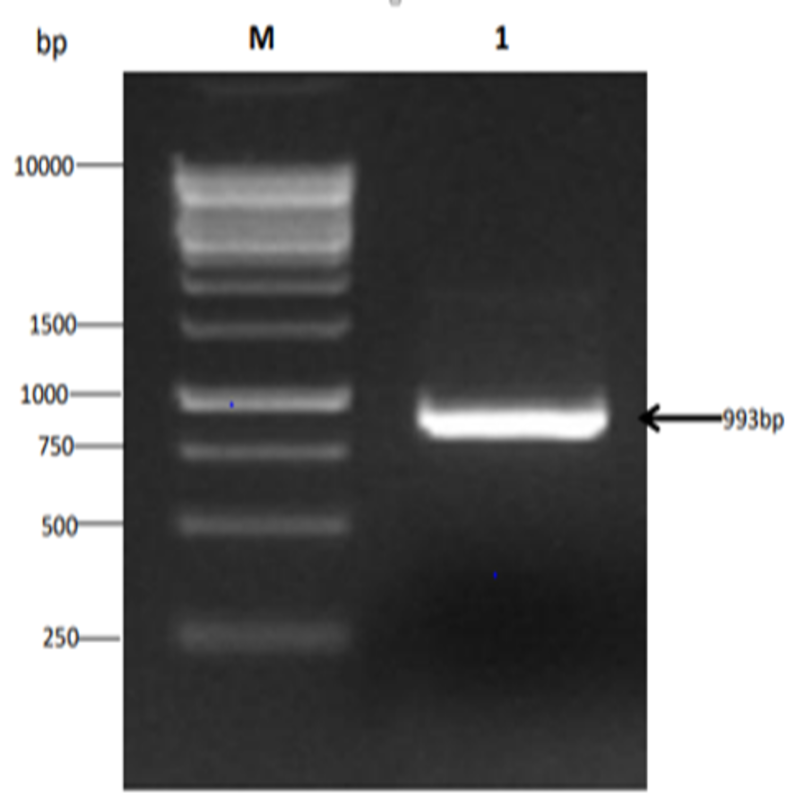

Supplement: S3 Fig — Lane M represents Thermo scientific Gene Ruler 1 kb DNA ladder for size comparison of amplified gene and Lane 1 represents DNA band of mutant endoglucanase B (993bp). (TIF) [file pone.0298716.s003.tif]

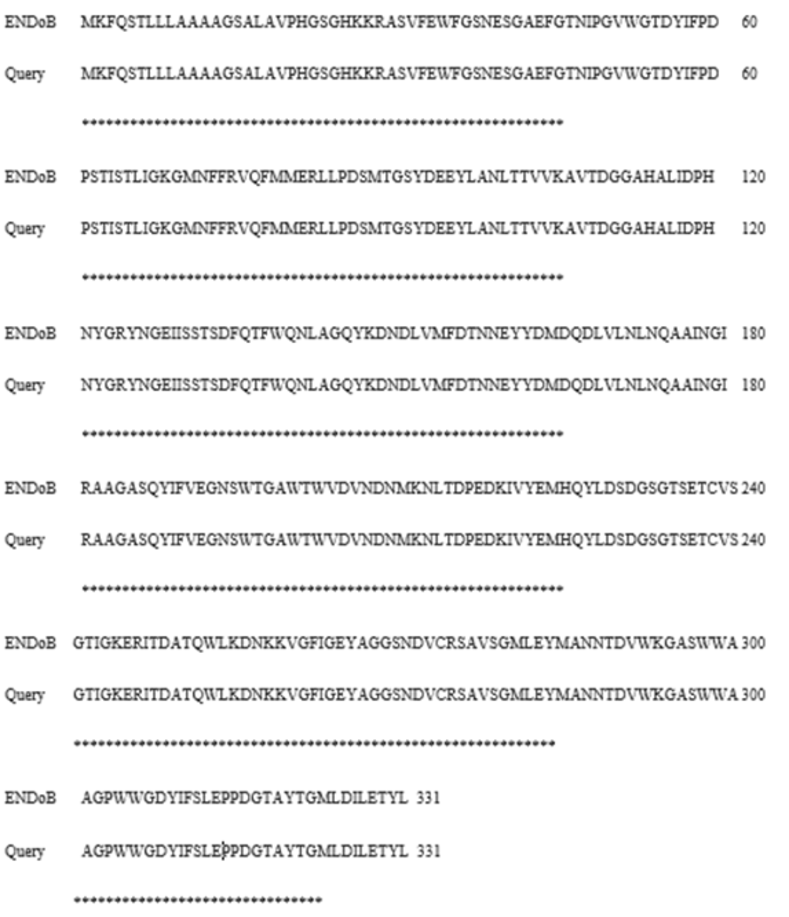

Supplement: S4 Fig — (TIF) [file pone.0298716.s004.tif]

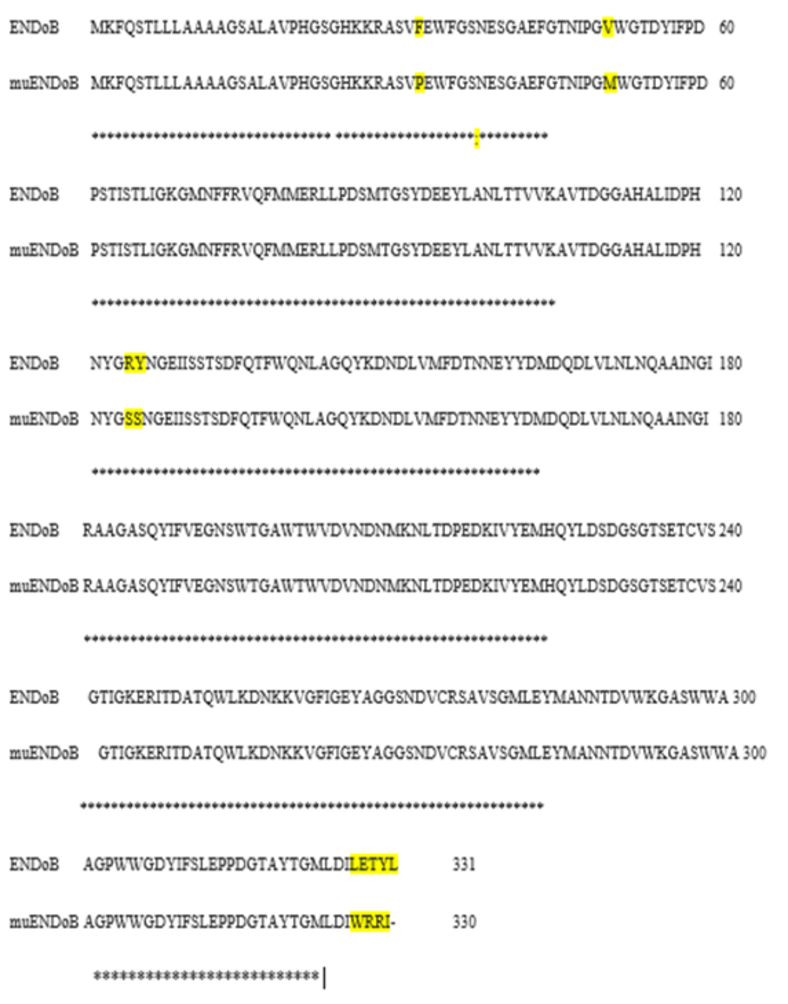

Supplement: S5 Fig — (TIF) [file pone.0298716.s005.tif]

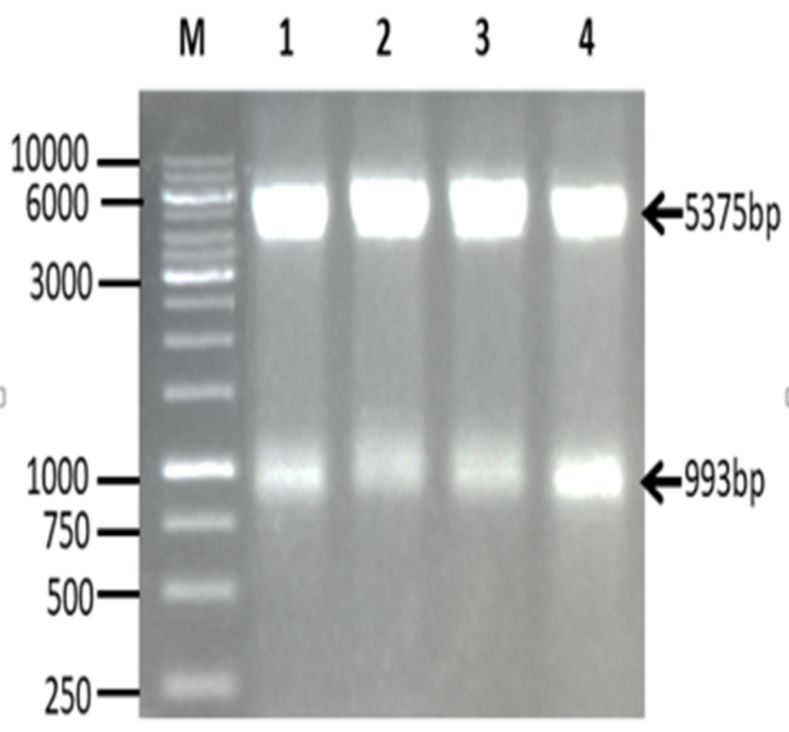

Supplement: S6 Fig — (TIF) [file pone.0298716.s006.tif]

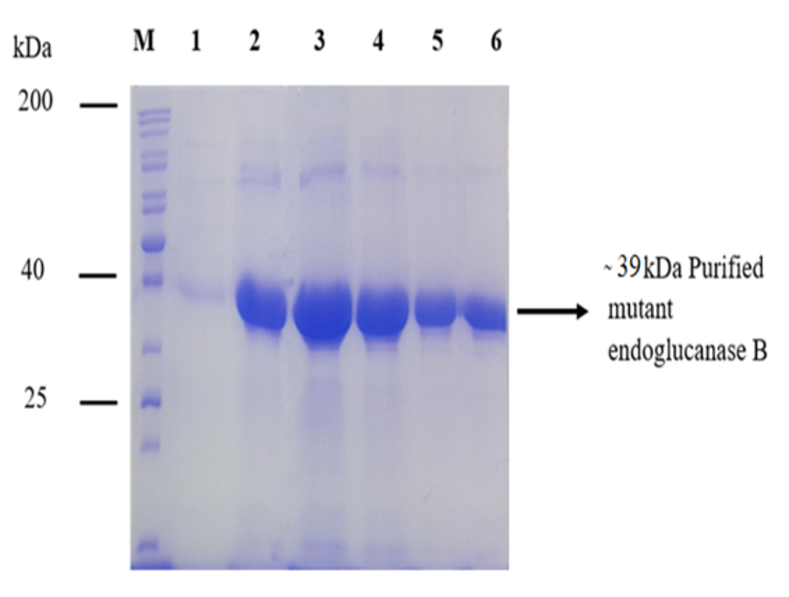

Supplement: S7 Fig — M: Mobilities of proteins of known molecular masses on SDS-PAGE, Lane 1–6: Fractions loaded from 2nd to 3rd of 200–250 mM of imidazole respectively. (TIF) [file pone.0298716.s007.tif]

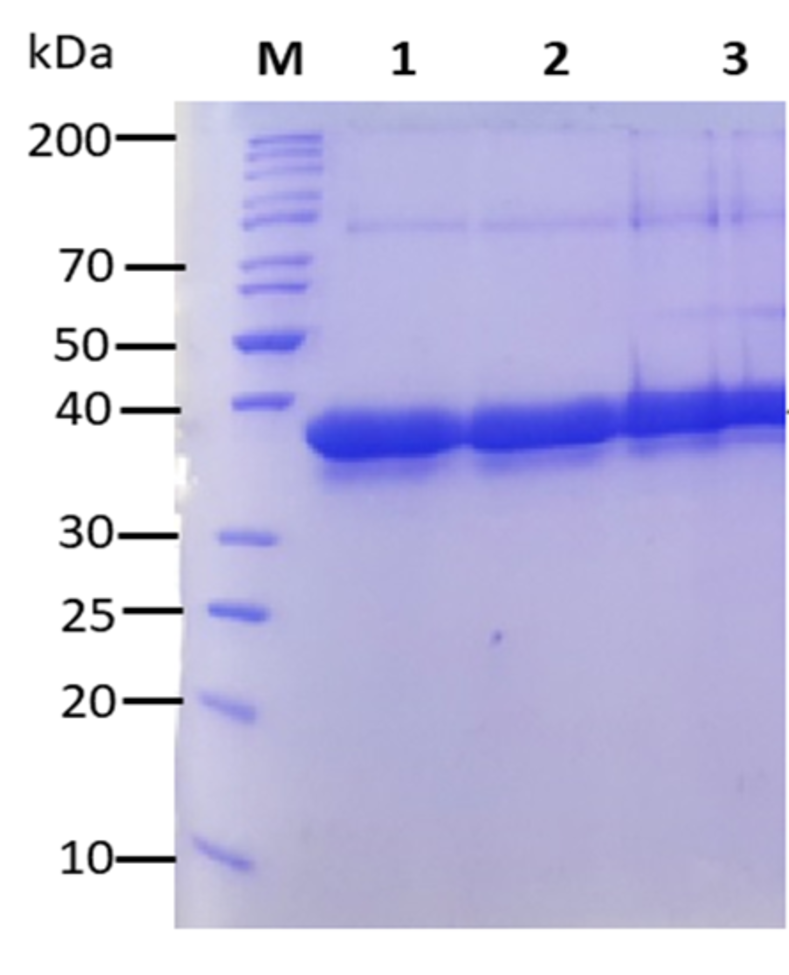

Supplement: S8 Fig — Lane M: Mobilities of proteins of known molecular masses on SDS-PAGE. Lane 1–3: showing purified dialysed fraction of endoglucanase B native. (TIF) [file pone.0298716.s008.tif]

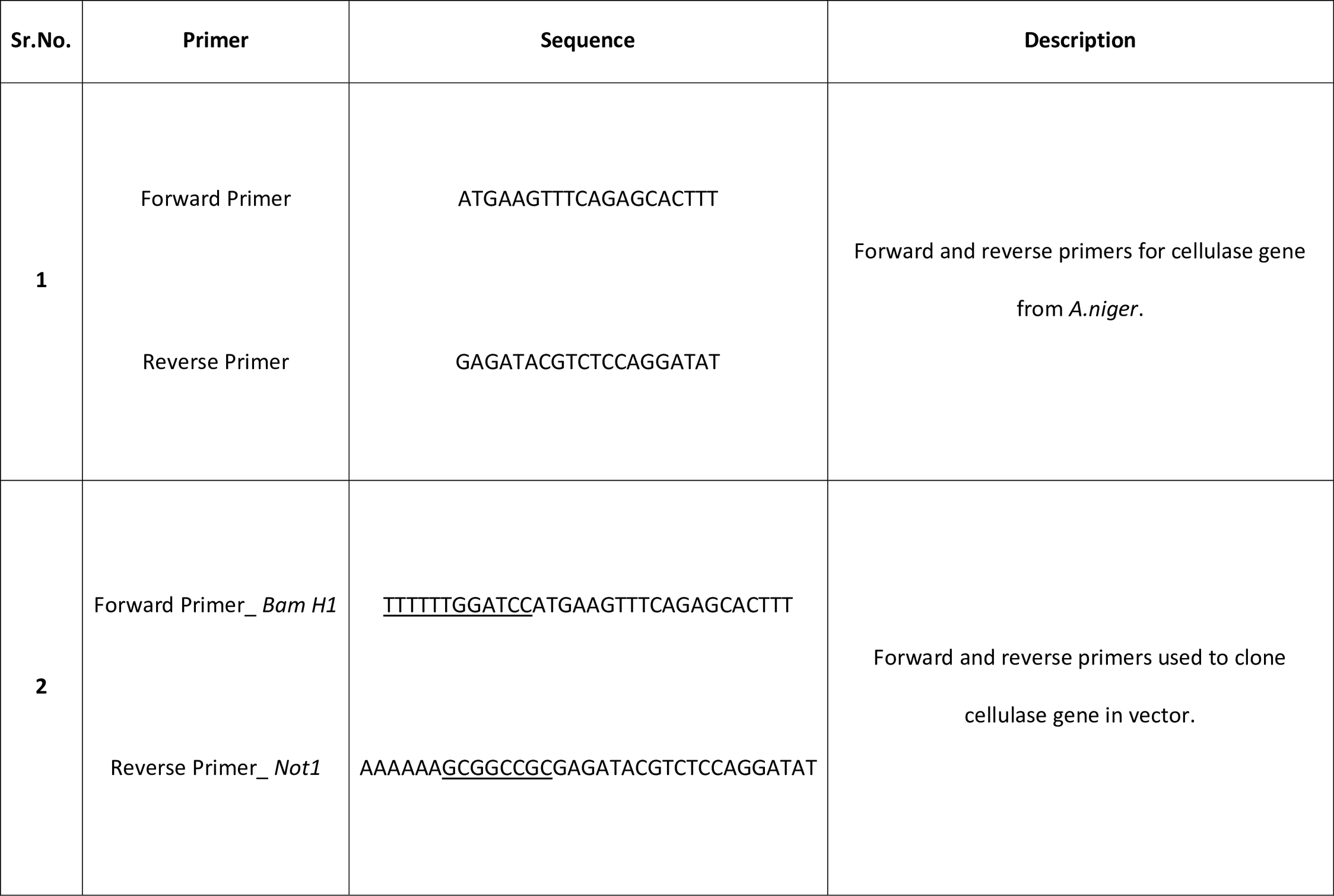

Supplement: S1 Table — (TIF) [file pone.0298716.s009.tif]
