# Supplementary material for: Heterologous expression and characterization of mutant cellulase from indigenous strain of Aspergillus niger
Source: PLoS One. 2024 May 15;19(5):e0298716. doi: 10.1371/journal.pone.0298716 (PMC11095671; doi:10.1371/journal.pone.0298716)
Supplement: Attachment — Submitted filename: Review Report of Paper.docx [file pone.0298716.s010.docx]

I have gone through the manuscript entitled, “Heterologous expression and characterization of mutant cellulase from indigenous strain of *Aspergillus niger*” and recommends the ACCEPTANCE of manuscript for the publication in PLOS One in view of following observations/minor corrections:

1. The manuscript describes an original research work elaborating expression of cellulase gene from an indigenous strain of *Aspergillus niger* and significantly contributes to the knowledge of microbial gene expressions for successful hyper expression of enzymes (Cellulase).
2. The experiments have been performed upto the mark and have been described in a detailed way.
3. Experimental conclusions are in accordance with the findings of the researchers.
4. Under sub heading; Solvent Accessible Surface Area (SASA), The statement describing stability of endoglucanase B is incomplete and it should be written in the past tense rather than present tense.
5. Same suggestion for the statement under sub heading; hydrogen bond analysis of wild and mutant type of endoglucanase B
6. On Page 28, there should be proper space among values of predicted ligand binding residues.
7. There is need to mention the version of Reprof software used for predicting wild type residues.
